# Modeling Complex Orthopedic Trauma in Rodents: Bone, Muscle and Nerve Injury and Healing

**DOI:** 10.3389/fphar.2020.620485

**Published:** 2021-02-01

**Authors:** Huaishuang Shen, Aysha M. Gardner, Juhee Vyas, Ryosuke Ishida, Vivianne L. Tawfik

**Affiliations:** ^1^ Department of Anesthesiology, Perioperative and Pain Medicine, Stanford University, Stanford, CA, United States; ^2^ Department of Orthopaedic Surgery, First Affiliated Hospital of Soochow University, Suzhou, China; ^3^ Department of Anesthesiology, Shimane University, Shimane, Japan; ^4^ Wu Tsai Neurosciences Institute, Stanford University, Stanford, CA, United States

**Keywords:** pain, chronic pain, translation, regeneration, preclinical

## Abstract

Orthopedic injury can occur from a variety of causes including motor vehicle collision, battlefield injuries or even falls from standing. Persistent limb pain is common after orthopedic injury or surgery and presents a unique challenge, as the initiating event may result in polytrauma to bone, muscle, and peripheral nerves. It is imperative that we understand the tissue-specific and multicellular response to this unique type of injury in order to best develop targeted treatments that improve healing and regeneration. In this Mini Review we will first discuss current rodent models of orthopedic trauma/complex orthotrauma. In the second section, we will focus on bone-specific outcomes including imaging modalities, biomechanical testing and immunostaining for markers of bone healing/turnover. In the third section, we will discuss muscle-related pathology including outcome measures of fibrosis, muscle regeneration and tensile strength measurements. In the fourth section, we will discuss nervous system-related pathology including outcome measures of pain-like responses, both reflexive and non-reflexive. In all sections we will consider parallels between preclinical outcome measures and the functional and mechanistic findings of the human condition.

## Introduction

High energy trauma is a major public health concern as it is often associated with complex muscle, bone, nerve and connective tissue damage. Military injuries to extremities, including those with extensive soft tissue and bone destruction, are on the rise (Stojadinovic et al., 2006) with the most frequent injury in the Iraq and Afghanistan wars being blast wounds (Belmont et al., 2016). In a cohort study of battle injuries, combat-related extremity injuries required longer hospital stays and were responsible for 65% of total inpatient resource utilization (Masini et al., 2009). These injuries cause pain and long-lasting functional deterioration which demand intensive medical intervention and physical therapy. The lifetime medical cost of non-fatal crash injuries, for example, was estimated to be $18.4 billion in 2012 (Bergen et al., 2014). It is therefore imperative that we develop better treatments to alleviate acute and chronic trauma-related pain and functional deficits to facilitate patient rehabilitation. Here, we review preclinical rodent orthopedic trauma/injury models, including discussion of clinical relevance and face validity of these models with respect to the human condition. We further discuss outcomes to evaluate bone, muscle and nerve healing that go beyond simple reflexive measures of nociception. While a substantial number of studies have demonstrated benefits of analgesic drugs in preclinical pain models, failure to translate these findings into clinically successful medications has resulted in shuttering analgesic drug development programs at several major companies (Percie du Sert and Rice, 2014). The reasons for these translational failures have been extensively reviewed elsewhere (Woolf, 2010; Barrett, 2015; Clark, 2016) and include poor animal models, poor pain measures and poor reporting practices. We therefore encourage investigators to consider clinically-informed models and outcomes as a means to bridge the gap between preclinical and clinical research efforts, particularly in the search for novel analgesics.

## Preclinical Models of Orthopedic Trauma

Preclinical animal research is key to uncovering mechanisms underlying traumatic injury. Although the small size of rodents makes standardized orthopedic injury models and outcome measurements quite challenging, the possibility of genetic manipulation renders rodent models, especially mice, the ideal species for many studies, but ultimately depends on the exact questions being asked (Jacenko and Olsen, 1995; Houdebine, 2007).

One of the first models of orthopedic trauma was developed by Bonnarens and Einhorn and involved closed femur fracture with intramedullary (IM) pinning in the rat (Bonnarens and Einhorn, 1984). Subsequently, Manigrasso and O’Connor (2004) described a mouse model for further molecular and genetic analysis. To more closely approximate human bone fixation, modifications have been reported by Holstein et al. (2007), with the use of a locking nail or compression screw (Holstein et al., 2009) to fix rotational and axial movement of the IM pin to avoid secondary injury from micromotion of the pin within the marrow space. In order to develop a model with better reproducibility and less tissue damage, closed tibial fracture models were also established (Hiltunen et al., 1993; Otto et al., 1995; Handool et al., 2018). External fixation was also utilized in rats by Mark et al. (2003) and further enhanced using customized fixators for studies focused on implanted osteoconductive materials (Kaspar et al., 2007). Additionally, different non-union rodent models were developed to study delayed healing or non-unions (Garcia et al., 2013). For example, atrophic non-union models that result from periosteal injury (Garcia et al., 2008) or segmental defect (Garcia et al., 2011), critical size defect models that cause reliable non-unions with proper fixation (Zwingenberger et al., 2013), and osteosynthesis-associated infections models (Wong et al., 2020) have all been adopted for mechanistic investigations.

When animal models accurately mimic the human condition, conclusions derived from their use have the potential to be effectively translated to clinical care. Orthopedic trauma is not only limited to bone injury, but also results in destruction of muscle, soft tissue and peripheral nerve damage that contribute to healing complications (Haffner-Luntzer et al., 2019). As a result, we developed a complex orthopedic trauma mouse model (Tawfik et al., 2020b), consisting of open tibial fracture with pin fixation and muscle injury, to reflect nociceptive sensitization, muscle fibrosis, and muscle fiber loss, as well as bone injury. Other examples of models that seek to more closely emulate the human condition include trauma-hemorrhage mouse models combining external femur fixator with pressure-controlled hemorrhagic shock on femoral arteries. Such models demonstrate that hemorrhagic shock can cause delayed fracture healing with decreased callus strength (Gascho et al., 1989; Neunaber et al., 2013; Bundkirchen et al., 2017). Similarly, femoral artery resection (Lu et al., 2007; Kalogeris et al., 2012) and diabetic ischemia (Follak et al., 2004; Kayal et al., 2009) have been combined with fracture models to mimic bleeding and ischemia in trauma patients (Miclau et al., 2017). Finally, the effects of osteoporosis have been mimicked in fracture models using ovariectomized female (Yousefzadeh et al., 2020), aged (Meyer et al., 2006) or transgenic mice with decreased bone density (Watanabe and Hishiya, 2005) to match clinical scenarios of postmenopausal and age-related osteoporosis, respectively.

## Pre-clinical Measurements in Orthopedic Trauma: Connection to the Clinic

**FIGURE 1:**
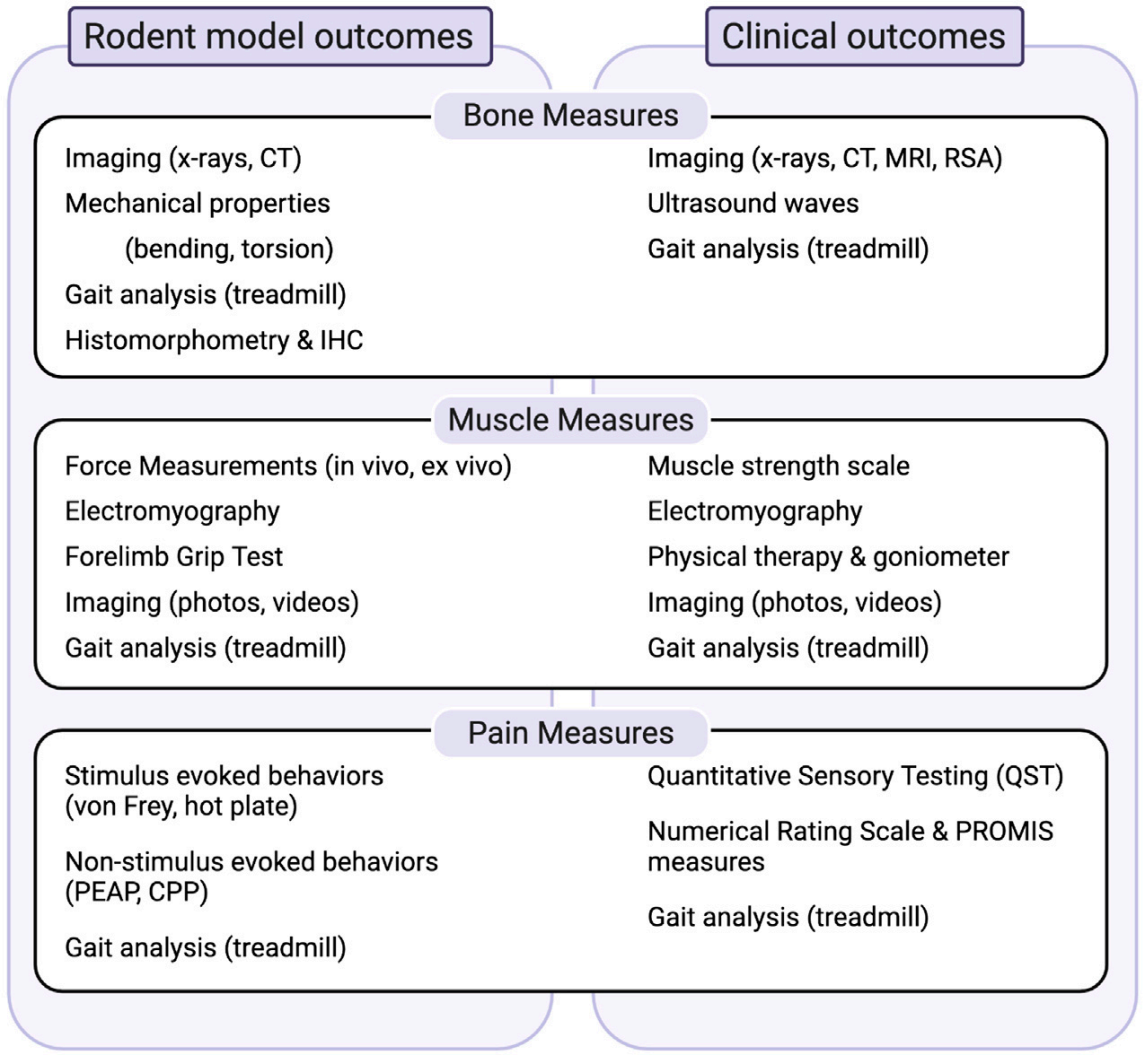
Summary of measures to evaluate bone, muscle and pain-related outcomes in preclinical models and their relationship to clinical outcomes. Abbrevations: CPP, conditioned pain preference; CT, computed tomography; IHC, immunohistochemistry; MRI, magnetic resonance imaging; PEAP, place escape/avoidance paradigms; PROMIS, patient reported outcomes measurement information systems; RSA, radiostereometric analysis.

Most important to the success of such models is the use of appropriate and translationally-relevant outcome measures with face validity with respect to the human condition. We describe the most widely used paradigms for evaluating bone and muscle healing as well as pain-like behaviors in rodent models of trauma with discussion of the clinical correlates that we believe enhance the likelihood of translation (Figure 1).

### Measurement of Bone Healing

#### Structural Analysis

Plain radiography (x-ray) is the most straightforward way to confirm bone bridge formation and post-operative displacement of implants or fixed bones (Schindeler et al., 2008; Manigrasso and O'Connor, 2010). However, detection of proper alignment alone without quantitative determination, does not provide full information about healing. In contrast to x-rays, micro-computed tomography (CT) is the most established imaging technique in rodent studies to examine bone structure, density, as well as fracture non-union (Schmidhammer et al., 2006). CT can be used to longitudinally monitor fracture healing at high resolution in living mice (Jiang et al., 2000). Using this highly accurate and consistent technique, researchers have reported differing geometric characteristics of long bones between sexes, strains, and ages of mice (Halloran et al., 2002; Ferguson et al., 2003; Willinghamm et al., 2010).

Although newly generated callus is a heterogeneous and three-dimensional structure, longitudinal or transverse bone sections are commonly stained with various dyes, including hematoxylin and eosin, safranin O/fast green and Masson’s trichrome stain etc., to differentiate cartilage, collagen and bone from smooth muscle (Miller et al., 2007; Hu et al., 2020). Besides qualitative evaluation, standardized histomorphometry can be used to assess bone healing quantitatively (Gerstenfeld et al., 2005). Additionally, immunohistochemistry can be used to detect changes of osteoblasts and osteoclasts, by alkaline phosphatase (ALP) and tartrate resistant acid phosphatase (TRAP) staining, respectively. Bone proteins, such as osteocalcin, bone morphogenetic proteins (BMPs), osteoprotegerin and vascular endothelial growth factor (VEGF), can be followed during the healing process from callus formation to bone resorption (Fedchenko and Reifenrath, 2014; Li et al., 2019; Li and Helms, 2021).

Physicians also rely heavily on imaging studies, including x-ray, CT, ultrasonography, and MRI (Lichtor et al., 1991; Cunningham et al., 2017), to evaluate anatomic bone healing. Specialized techniques, including radiostereometric analysis (RSA), further allow for precise radiographic measurement of fracture micromotion or deformation (Solomon et al., 2010).

#### Mechanical Properties

Most rodent studies use bending and torsion on long bones to mimic the typical loading modes in patients (Fritton et al., 2000). Monotonic bending is a major whole-bone measurement of bone ductility and can be performed in three-point or four-point bending (Liodaki et al., 2017; Zhang et al., 2017; Mumtaz et al., 2020; Zhang et al., 2020). While torsion tests are often used to evaluate fracture healing, fracture toughness tests (Poundarik et al., 2012), time-dependent tests (Lynch and Silva, 2008) and viscoelastic tests (Maruyama et al., 2015) are also common in fracture studies, as they are more sensitive to bone matrix changes. To measure the local properties of bone, nanoindentation is utilized to measure the moduli and hardness in different regions of the leg among different strains (Pathak et al., 2012; Pepe et al., 2020). Not only can this technique be applied to measure small biomaterial or callus tissue in rodent studies, but may also measure intrinsic properties of bone tissue in clinical studies by means of *trans*-iliac biopsy specimens (Vennin et al., 2017). Ultrasound waves can be used to non-invasively detect precise changes in both material density and structural integrity via velocity and attenuation in humans. For example, Sakai et al. (2008) used an echo-tracking system to evaluate patients’ bone strength under three-point bending tests. Factors affecting long bone fractures including body size (Jepsen et al., 2015), location (Schriefer et al., 2005), sex (Glatt et al., 2007), age (Brodt and Silva, 2010), and strain (Fritton et al., 2000) have been systematically summarized by Jepsen et al. (2015), along with practical guidelines on establishing biomechanical mechanisms in different situations.

#### Gait Analysis

Besides pain control and proper tissue healing, the ultimate goal of treatment for orthopedic injury is to restore the function of fractured limbs. For lower extremity injury, gait analysis has been frequently used as a measure of function in large animals (Muybridge, 1979; Seebeck et al., 2005), and in rodent models of nerve injury (Wang et al., 2008) or osteoarthritis (Williams et al., 1993), and in our own studies using the fracture-pin model of orthopedic trauma (Tawfik et al., 2020a).

Gait analysis using a treadmill can evaluate both static and dynamic gait patterns in mice by placing the rodent on a treadmill with a camera recording from below. This can be conducted with DigiGait or CatWalk (Kappos et al., 2017) analysis systems as described previously (Deuis et al., 2017). Based on Chen's modified systematic method (Chen et al., 2017), Hofman et al. (2020) evaluated five gait parameters including intensity, print area, swing speed, stand duration, duty cycle after intramedullary stabilized femoral fracture at multiple time points. Gait analysis can also be measured via a simple footprint test with paws covered in non-toxic paint and the pattern evaluated (Deuis et al., 2017).

As for clinical outcomes, gait analysis is an essential aspect of rehabilitation, especially for patients with lower extremity fractures (Rosenbaum et al., 2014). Macri et al. (Macri et al., 2012) proposed a standardized gait pattern score system in tibial fracture patients treated with an intramedullary nail that enables the classification stages of fracture consolidation. In the clinical setting, physicians and physical therapists can observe patients walking on the floor or on a treadmill to monitor gait, favoring of one limb over the other, or any changes in foot placement or asymmetry (Higginson, 2009).

#### Cellular Mechanisms of Bone Regeneration and Repair

**FIGURE 2:**
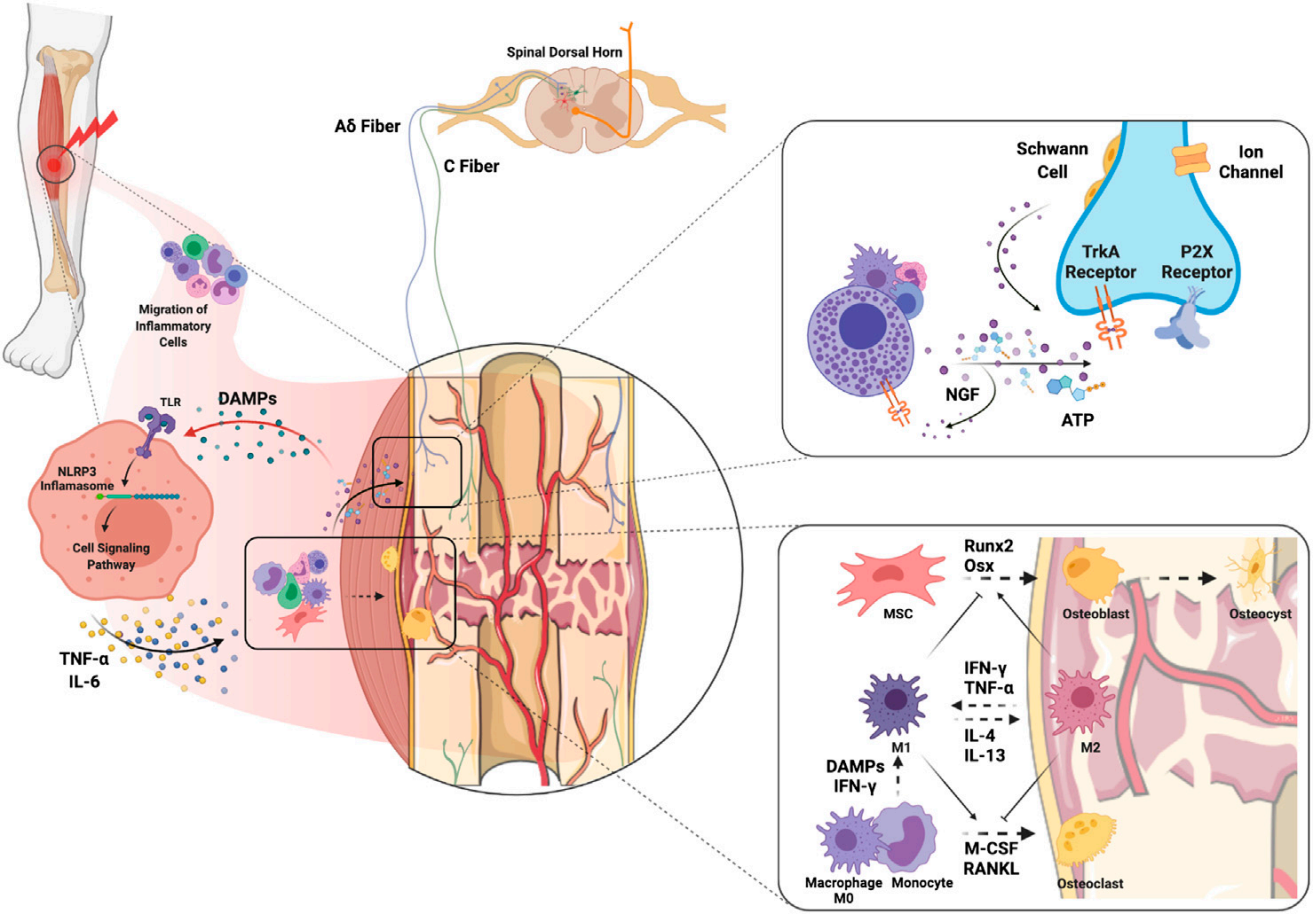
Molecular and cellular mechanisms of complex orthopedic trauma and recovery. Initial trauma results in inflammatory cell migration to the site of injury as a result of cytokine and chemokine release from injured cells. DAMPs released from injured muscle bind TLRs on immune cells which leads to NLRP3 inflammasome-mediated release of inflammatory cytokines such as NGF, TNF-α and IL-6. ATP released from immune cells and injured nerves themselves can activate P2X receptors on sensory nerve terminals. In addition, release of NGF from inflammatory cells stimulates TrkA receptors on Aδ and C fibers which can maintain hyperexcitability. NGF-TrkA complexes are transferred to the DRG where primary afferent cell bodies reside, and these complexes can further maintain pain. Macrophages interfacing with bone are polarized to the M1 phenotype by DAMPs and pro-inflammatory cytokines to clean necrotic tissue and any pathogens. The transition from M1 to M2 would encourage activation of MSCs and differentiation of osteoblasts to promote osteogenesis. The balance between osteoblastic and osteoclastic activity plays a key role in the bone remodeling that results in fracture healing. Abbreviations: DAMPs, damage-associated molecular patterns; IFN-γ, interferon-gamma; IL, interleukin; M-CSF, monocyte-colony stimulating factor; MSC, muscle stem cells; NGF, nerve growth factor; Osx, osterix; RANKL, receptor activator of nuclear factor kappa-B ligand; TLR, toll-like receptor; TNF-α, tumor necrosis factor alpha.

The mechanisms of bone healing have been widely studied and recently comprehensively reviewed by Loi et al. (2016). It is important to highlight that inflammatory cells and their mediators both contribute to bone healing in the early stages but can also delay fracture union if inflammation becomes persistent (Figure 2). In particular, macrophages participate in bone fracture healing immediately after injury when the M1 “pro-inflammatory” subtype predominates and encourages greater mineralization through interactions with osteoprogenitors and mesenchymal stem cells (MSCs, Figure 2) (Loi et al., 2016). Yet persistent macrophage activation and proinflammatory cytokine expression is detrimental to proper bone healing by inhibiting the differentiation of MSCs to osteoblasts (Lin et al., 2017), and can be a trigger for peripheral neuron sensitization (Shepherd et al., 2018). Polarization of M1 macrophages to the M2 “anti-inflammatory” phenotype using IL-4 infusion or MSC injection in the subacute period favors bone regeneration and presents an attractive approach to enhance fracture healing (Lin et al., 2018; Lin et al., 2019). In addition, osteocytes have important roles in every phase of fracture healing as they can sense both physical and biochemical signals to regulate bone metabolism regeneration and remodeling (Bonewald, 2011). For an in-depth review of the important role of osteocytes in bone healing see Choy et al. (2020). Bone healing occurs in the context of revascularization and reinnervation which are integral to the process. For example, with implants containing spatiotemporally released angiogenic factors, (Freeman et al. 2020) accelerated bone healing in a large bone defect mouse model. Moreover, Li et al. (2019) demonstrated that the neuronal NGF receptor, TrkA, is a key upstream mediator in ulnar stress fracture healing, clearly connecting neuronal responses with bone repair (also see *Molecular Mechanisms of Pain After Orthopedic Trauma* section).

### Measurement of Muscle Healing

#### Muscle Force

Measurement of muscle force is important to determine the extent of strength regained after injury. Viscoelastic force relaxation, twitch dynamics, and max tetanic force measurements are performed by exposing the sciatic nerve and tibialis anterior (TA) and attaching them to a force transducer (Quarta et al., 2018). *Ex-vivo* measurements of muscle dynamics are performed by removing the TA muscle and attaching it to a force transducer lever and the testing apparatus (Quarta et al., 2017). The contraction of the TA is electrically induced through immersion in a culture bath, while force production was measured (Quarta et al., 2017). In the same way, isokinetic muscle functioning testing can record the forces applied by muscle groups in humans (Osternig, 1986).

#### Muscle Innervation/Electrical Conduction

In both mouse models and human injuries, electromyography (EMG) is used to diagnose and evaluate the prognosis of myopathy (Daube and Rubin, 2009) while also examining muscle innervation. Researchers surgically put the EMG electrodes within the muscle of interest in mice, then electrically stimulate the treated muscle and record the induced peak-to-peak voltage response (Sicari et al., 2014). Similarly, clinicians place needle electrodes in the belly of injured muscle and motor nerve conduction amplitude pre- and post-treatment can be recorded (Dziki et al., 2016).

#### Muscle Strength/Function

Several testing paradigms have been developed to evaluate muscle strength and function in rodents with clear clinical correlates. The forelimb grip test evaluates a mouse’s forelimb and/or hindlimb skeletal muscle strength (Bonetto et al., 2015). These compare to the 1–5 scale from the British Medical Research Council for muscle strength testing in humans (Compston, 2010). Physical therapists also use a goniometer to collect range of motion measurements on the injured limb (Grogan et al., 2011). Lastly, photos and videos of injured and surrounding muscles are used for mice models and humans to evaluate functional movements and to record the wounds and atrophy of the muscle (Grogan et al., 2011).

Exercise with either wheel or treadmill access is an additional way to measure motor function and provide rehabilitation after injury in mice. Shi et al. (2018) demonstrated that 4 weeks of exercise resolved allodynia, warmth, swelling, and unweighting compared to unexercized mice in a fracture-casting model. Our laboratory has also shown the effectiveness of delaying exercise. In the tibial fracture-pin model of orthopedic trauma, mice that had access to a running wheel immediately after injury had worse muscle fibrosis compared to non-exercized mice, however, mice with delayed access to exercise had improved muscle, bone and pain outcomes (Tawfik et al., 2020b). Routine exercise can prove to be extremely beneficial for patients by improving function and decreasing pain (Merkle et al., 2020).

Monitoring mice throughout their daily activity using systems such as HomeCageScan which utilize automated video can give a better sense of spontaneous pain behaviors and movement (Deuis et al., 2017). Similarly, tracking daily movement using a body-fixed sensor can facilitate monitoring of activities of daily living (ADL) and post-op rehabilitation in patients (Brandes et al., 2011; Benzinger et al., 2014). Luna et al. (2019) used actigraphy-based assessments to evaluate early postoperative physical function. They correlated intensity of activity to individual recovery trajectories and found that early stratified physiotherapy interventions are needed for patients with reduced activity.

#### Cellular Mechanisms of Muscle Regeneration and Repair

After a muscle injury, the skeletal muscle regenerates via activation, proliferation, migration, and differentiation of muscle stem cells in a conducive microenvironment (Sicari et al., 2014). It has also been shown that perivascular stem cells, CD146^+^ endothelial cells, and NG2^+^ (neurogenin 2-positive) polydendrocytes, and M1 and M2 macrophages have a crucial role in muscle regeneration (Sicari et al., 2014; Hurtgen et al., 2016). Without correct muscle repair, volumetric muscle loss (VML) can ensure with associated chronic muscle pain, therefore there is a need to develop new therapies to ensure proper muscle regeneration (Figure 2).

Quarta et al. (2017) used bioconstructs of muscle stem cells and other muscle resident cells to restore structure and function after an acute VML. To further support muscle regeneration, Sicari et al. (2014) implanted a porcine urinary bladder extracellular matrix (ECM) into VML injured muscle to create a supportive microenvironmental niche. Moreover, they performed successful proof-of-concept studies in five patients with VML injury (Sicari et al., 2014) and a subsequent trial with thirteen patients (Dziki et al., 2016) also showed strength increase, *de novo* muscle formation, and muscle innervation. Clear limitations include the small number of participants, possible placebo effect, lack of a control group, and not being able to correlate *de novo* muscle formation to functional improvement, however, these are steps toward the clinical use of such bioconstructs and ECM transplantation for VML.

### Measurement of Pain-like Behaviors

#### Reflexive Behaviors

Reflexive stimulus-evoked measures with varying modalities (touch, temperature, pressure) are commonly used approaches to assess pain-like behaviors in preclinical models. Tests used for mechanically evoked pain-like behaviors include manual or electronic von Frey test, and Randall-Selitto test (Deuis et al., 2017). Tests used to evaluate heat responses include the hot-plate, Hargreaves, and thermal probe tests (Deuis et al., 2017). In humans, mechanical allodynia and hyperalgesia can be assessed using a pinprick or monofilament, or through the application of pressure while heat sensitivity can be assessed by placing a metal probe on the skin (Deuis et al., 2017). Quantitative Sensory Testing (QST), dynamic or static, can be used to evaluate stimulus-evoked pain. Dynamic QST assesses response to multiple stimuli and allows for examination of central processing of nociception, whereas static QST assesses the response to a single stimulus (Mackey et al., 2017). Dynamic QST includes temporal summation (TS) and conditioned pain modulation (CPM). In TS, continual probing by noxious stimuli causes increased perception of pain, whereas in CPM the perception of pain caused by a noxious stimulus can be decreased with the presence of a second noxious stimulus (Mackey et al., 2017).

#### Non-Reflexive Behaviors

Methods used to assess non-reflexive pain-related behaviors include gait analysis (see above), and place escape/avoidance paradigms (PEAP) and conditioned place preference (CPP) (Navratilova and Porreca, 2014). PEAP assesses the affective and sensory components of pain while CPP facilitates understanding of reward and aversion behavior motivated by pain-relief, or pain avoidance (Navratilova and Porreca, 2014; Kuhn et al., 2019). For example, a change in escape latency in the Mechanical Conflict Avoidance test (MCA)–a voluntary, non-reflexive behavioral assay–could indicate spontaneous pain after spared nerve injury or Complete Freund's adjuvant injection (Shepherd and Mohapatra, 2018). The mouse grimace scale (MGS) is another measure of spontaneous pain. The MGS scores the movements of individual facial muscles on a three-point rating scale. It accounts for orbital tightening, cheek bulge, nose bulge, whisker position, and ear position (Mogil et al., 2020).

The Numerical Rating Scale (NRS) is frequently used to assess pain in humans but does not provide much depth of information. It allows patients to rate pain on a scale of 0–10 (or 0–100), with the lower limit indicating no pain and the higher limit denoting the worst pain (Krebs et al., 2007). Other more detailed evaluations were subsequently developed to more fully evaluate patients presenting with chronic pain. The McGill Pain Questionnaire, for example, is a multidimensional framework that measures the sensory, affective, cognitive, and behavioral aspects that comprise pain by evaluating pain location, intensity, quality, and pattern (Ngamkham et al., 2012). More recently, several pain clinics including our own (Bhandari et al., 2016), have integrated patient-reported outcome (PROMIS) measures that evaluate physical, psychological and social functioning, fatigue and sleep, among other parameters (Sturgeon et al., 2015a; Sturgeon et al., 2015b).

#### Molecular Mechanisms of Pain After Orthopedic Trauma

The bone periosteum, made up of outer fibrous and inner cambium layers, appears to have the densest sensory and sympathetic innervation (Mantyh, 2014). Pain following bone fracture is attributed to the nerve fibers in the periosteum, including A-delta and C-fibers which get sensitized after injury (Mantyh, 2014). Initial activation of neuronal P2X receptors by ATP released from injured keratinocytes and infiltrating inflammatory cells facilitates the transmission of nociceptive information through membrane depolarization and calcium entry (Bernier et al., 2018). The inflammatory response to injury further results in the release of nerve growth factor (NGF) by immune and Schwann cells, which binds to TrkA (Mantyh et al., 2011). NGF-TrkA form a complex which is internalized and transported to cell bodies in the DRG where feedforward hypersensitivity of neurons triggers pain (Mantyh et al., 2011). Since the NGF-TrkA complex can generate and maintain pain, the use of anti-NGF, which prevents the binding of NGF to TrkA, can reduce pain-related behaviors in a fracture model without negatively affecting bone healing (Jimenez-Andrade et al., 2007; Koewler et al., 2007). In clinical trials, the administration of tanezumab, a monoclonal antibody directed against NGF, was reported to exhibit efficacy in low back pain (Chang et al., 2016).

When a peripheral injury occurs, endogenous danger-associated molecular patterns (DAMPs) are released from damaged muscle fibers to facilitate repair (Hurtgen et al., 2016; Kelley et al., 2019). DAMPs trigger an innate immune response to injury by binding to pattern recognition receptors, such as toll-like receptors (TLRs) (Bianchi, 2007) with downstream activation of the NLRP3 inflammasome and production of inflammatory mediators such as TNF-alpha and IL-6, among others (Bianchi, 2007) (Figure 2). TNF-alpha and IL-6 have both been shown to cause hyperalgesia when intramuscularly injected into rodents likely through induction of additional sensitizing mediators such as PGE_2_, NGF, and CGRP (Schafers et al., 2003; Manjavachi et al., 2010). Importantly, commonly used clinical medications such as dexamethasone and morphine can prevent the IL-6-induced reduction in pain threshold (Manjavachi et al., 2010). While these drugs can provide some relief for post-injury pain, there is still a need to identify better treatment options (Manjavachi et al., 2010).

#### Imaging Pain Generators

One way to identify pain generators is through the use of positron emission tomography (PET), a noninvasive imaging tool that allows spatiotemporal visualization of cellular responses to injury. PET ligands that specifically target different cell types and varying cellular activation states can be utilized to monitor disease progression or measure treatment success (Jain et al., 2020). We previously used longitudinal ^18^F-TSPO-PET imaging in the tibial fracture mouse model to track the activation of peripheral and central myeloid cells during the acute-to-chronic pain transition (Cropper et al., 2019). In patients, we have also used PET/MRI imaging with the novel radiotracer ^18^F-FTC-146 to image the sigma-1 receptor, a pro-nociceptive receptor which is upregulated in inflamed tissue. Imaging identified a previously unappreciated mass with high focal uptake of ^18^F-FTC-146 in the intercondylar notch, removal of which resulted in resolution of the patient's knee pain (Cipriano et al., 2018).

## Conclusions and Future Directions

Mouse models of orthopedic trauma that mimic multi-tissue injury, including bone, muscle and nerve, are most representative of human complex extremity trauma. Using such models combined with outcome measures that have clear human correlates, will improve translation of basic science findings to clinically useful treatments. We encourage all preclinical researchers to consider these approaches in designing their studies.

## Author Contributions

All authors (HS, AG, JV, RI, and VT) contributed to the formulation of the ideas for this review and to the writing and editing of the manuscript and figures. All authors approved the final version.

## Funding

This work was supported by grant funds from the Department of Anesthesiology, Perioperative and Pain Medicine Stanford University School of Medicine (VT) and National Institutes of Health (NIH) Grant No: R35 GM137906 (VT). Individual fellowship support was provided by the Department of Orthopaedic Surgery, First Affiliated Hospital of Soochow University (HS) and the Department of Anesthesiology, Shimane University (RI).

## Conflict of Interest

The authors declare that the research was conducted in the absence of any commercial or financial relationships that could be construed as a potential conflict of interest.

## References

[B1] Barrett J. E. (2015). The pain of pain: challenges of animal behavior models. Eur. J. Pharmacol. 753, 183–190. 10.1016/j.ejphar.2014.11.046 25583180

[B2] Belmont P. J. Owens B. D. Schoenfeld A. J. (2016). Musculoskeletal injuries in Iraq and Afghanistan: epidemiology and outcomes following a decade of war. J. Am. Acad. Orthop. Surg. 24 (6), 341–348. 10.5435/JAAOS-D-15-00123 27115793

[B3] Benzinger P. Lindemann U. Becker C. Aminian K. Jamour M. Flick S. E. (2014). Geriatric rehabilitation after hip fracture. Role of body-fixed sensor measurements of physical activity. Z. Gerontol. Geriatr. 47 (3), 236–242. 10.1007/s00391-013-0477-9 23780628

[B4] Bergen G. Peterson C. Ederer D. Florence C. Haileyesus T. Kresnow M. J. (2014). Vital signs: health burden and medical costs of nonfatal injuries to motor vehicle occupants - United States, 2012. Morb. Mortal. Wkly. Rep. 63 (40), 894–900. PMC4584612 25299606

[B5] Bernier L. P. Ase A. R. Séguéla P. (2018). P2X receptor channels in chronic pain pathways. Br. J. Pharmacol. 175 (12), 2219–2230. 10.1111/bph.13957 28728214 PMC5980614

[B6] Bhandari R. P. Feinstein A. B. Huestis S. E. Krane E. J. Dunn A. L. Cohen L. L. (2016). Pediatric-Collaborative Health Outcomes Information Registry (Peds-CHOIR): a learning health system to guide pediatric pain research and treatment. Pain 157 (9), 2033–2044. 10.1097/j.pain.0000000000000609 27280328 PMC4988911

[B7] Bianchi M. E. (2007). DAMPs, PAMPs and alarmins: all we need to know about danger. J. Leukoc. Biol. 81 (1), 1–5. 10.1189/jlb.0306164 17032697

[B8] Bonetto A. Andersson D. C. Waning D. L. (2015). Assessment of muscle mass and strength in mice. Bonekey Rep. 4, 732 10.1038/bonekey.2015.101 26331011 PMC4549925

[B9] Bonewald L. F. (2011). The amazing osteocyte. J. Bone Miner. Res. 26 (2), 229–238. 10.1002/jbmr.320 21254230 PMC3179345

[B10] Bonnarens F. Einhorn T. A. (1984). Production of a standard closed fracture in laboratory animal bone. J. Orthop. Res. 2 (1), 97–101. 10.1002/jor.1100020115 6491805

[B11] Brandes M. Ringling M. Winter C. Hillmann A. Rosenbaum D. (2011). Changes in physical activity and health-related quality of life during the first year after total knee arthroplasty. Arthritis Care Res. (Hoboken). 63 (3), 328–334. 10.1002/acr.20384 20981812

[B12] Brodt M. D. Silva M. J. (2010). Aged mice have enhanced endocortical response and normal periosteal response compared with young-adult mice following 1 week of axial tibial compression. J. Bone Miner. Res. 25 (9), 2006–2015. 10.1002/jbmr.96 20499381 PMC3153404

[B13] Bundkirchen K. Macke C. Reifenrath J. Schäck L. M. Noack S. Relja B. (2017). Severe hemorrhagic shock leads to a delayed fracture healing and decreased bone callus strength in a mouse model. Clin. Orthop. Relat. Res. 475 (11), 2783–2794. 10.1007/s11999-017-5473-8 28795328 PMC5638746

[B14] Chang D. S. Hsu E. Hottinger D. G. Cohen S. P. (2016). Anti-nerve growth factor in pain management: current evidence. J. Pain Res. 9, 373–383. 10.2147/JPR.S89061 27354823 PMC4908933

[B15] Chen H. Du J. Zhang Y. Barnes K. Jia X. (2017). Establishing a reliable gait evaluation method for rodent studies. J. Neurosci. Methods 283, 92–100. 10.1016/j.jneumeth.2017.03.017 28351803 PMC5461654

[B16] Choy M. H. V. Wong R. M. Y. Chow S. K. H. Li M. C. Chim Y. N. Li T. K. (2020). How much do we know about the role of osteocytes in different phases of fracture healing? A systematic review. J. Orthop. Translat. 21, 111–121. 10.1016/j.jot.2019.07.005 32309136 PMC7152791

[B17] Cipriano P. W. Lee S. W. Yoon D. Shen B. Tawfik V. L. Curtin C. M. (2018). Successful treatment of chronic knee pain following localization by a sigma-1 receptor radioligand and PET/MRI: a case report. J. Pain Res. 11, 2353–2357. 10.2147/JPR.S167839 30349360 PMC6190812

[B18] Clark J. D. (2016). Preclinical pain research: can we do better? Anesthesiology 125 (5), 846–849. 10.1097/ALN.0000000000001340 27575448 PMC5176336

[B19] Compston A. (2010). Aids to the investigation of peripheral nerve injuries. Medical Research Council: nerve Injuries Research Committee. His Majesty's Stationery Office: 1942; pp. 48 (iii) and 74 figures and 7 diagrams; with aids to the examination of the peripheral nervous system. By Michael O'Brien for the Guarantors of Brain. Saunders Elsevier: 2010; pp. [8] 64 and 94 Figures. Brain 133 (10), 2838–2844. 10.1093/brain/awq270 20928945

[B20] Cropper H. C. Johnson E. M. Haight E. S. Cordonnier S. A. Chaney A. M. Forman T. E. (2019). Longitudinal translocator protein-18 kDa-positron emission tomography imaging of peripheral and central myeloid cells in a mouse model of complex regional pain syndrome. Pain 160 (9), 2136–2148. 10.1097/j.pain.0000000000001607 31095093 PMC6527343

[B21] Cunningham B. P. Brazina S. Morshed S. Miclau T. 3rd (2017). Fracture healing: a review of clinical, imaging and laboratory diagnostic options. Injury 48 (Suppl. 1), S69–S75. 10.1016/j.injury.2017.04.020 28483359

[B22] Daube J. R. Rubin D. I. (2009). Needle electromyography. Muscle Nerve 39 (2), 244–270. 10.1002/mus.21180 19145648

[B23] Deuis J. R. Dvorakova L. S. Vetter I. (2017). Methods used to evaluate pain behaviors in rodents. Front. Mol. Neurosci. 10, 284 10.3389/fnmol.2017.00284 28932184 PMC5592204

[B24] Dziki J. Badylak S. Yabroudi M. Sicari B. Ambrosio F. Stearns K. (2016). An acellular biologic scaffold treatment for volumetric muscle loss: results of a 13-patient cohort study. NPJ Regen. Med. 1, 16008 10.1038/npjregenmed.2016.8 29302336 PMC5744714

[B25] Fedchenko N. Reifenrath J. (2014). Different approaches for interpretation and reporting of immunohistochemistry analysis results in the bone tissue–a review. Diagn. Pathol. 9, 221 10.1186/s13000-014-0221-9 25432701 PMC4260254

[B26] Ferguson V. L. Ayers R. A. Bateman T. A. Simske S. J. (2003). Bone development and age-related bone loss in male C57BL/6J mice. Bone 33 (3), 387–398. 10.1016/s8756-3282(03)00199-6 13678781

[B27] Follak N. Klöting L. Wolf E. Merk H. (2004). Delayed remodeling in the early period of fracture healing in spontaneously diabetic BB/OK rats depending on the diabetic metabolic state. Histol. Histopathol. 19 (2), 473–486. 10.14670/HH-19.473 15024708

[B28] Freeman F. E. Pitacco P. van Dommelen L. H. A. Nulty J. Browe D. C. Shin J. Y. (2020). 3D bioprinting spatiotemporally defined patterns of growth factors to tightly control tissue regeneration. Sci. Adv. 6 (33), eabb5093 10.1126/sciadv.abb5093 32851179 PMC7428335

[B29] Fritton S. P. McLeod K. J. Rubin C. T. (2000). Quantifying the strain history of bone: spatial uniformity and self-similarity of low-magnitude strains. J. Biomech. 33 (3), 317–325. 10.1016/s0021-9290(99)00210-9 10673115

[B30] Garcia P. Herwerth S. Matthys R. Holstein J. H. Histing T. Menger M. D. (2011). The LockingMouseNail—a new implant for standardized stable osteosynthesis in mice. J. Surg. Res. 169 (2), 220–226. 10.1016/j.jss.2009.11.713 20371084

[B31] Garcia P. Histing T. Holstein J. H. Klein M. Laschke M. W. Matthys R. (2013). Rodent animal models of delayed bone healing and non-union formation: a comprehensive review. Eur. Cell. Mater. 26, 1–12. 10.22203/ecm.v026a01 23857280

[B32] Garcia P. Holstein J. H. Maier S. Schaumlöffel H. Al-Marrawi F. Hannig M. (2008). Development of a reliable non-union model in mice. J. Surg. Res. 147 (1), 84–91. 10.1016/j.jss.2007.09.013 18061614

[B33] Gascho J. A. Fanelli C. Zelis R. (1989). Aging reduces venous distensibility and the venodilatory response to nitroglycerin in normal subjects. Am. J. Cardiol. 63 (17), 1267–1270. 10.1016/0002-9149(89)90188-4 2496597

[B34] Gerstenfeld L. C. Wronski T. J. Hollinger J. O. Einhorn T. A. (2005). Application of histomorphometric methods to the study of bone repair. J. Bone Miner. Res. 20 (10), 1715–1722. 10.1359/JBMR.050702 16160729

[B35] Glatt V. Canalis E. Stadmeyer L. Bouxsein M. L. (2007). Age-related changes in trabecular architecture differ in female and male C57BL/6J mice. J. Bone Miner. Res. 22 (8), 1197–1207. 10.1359/jbmr.070507 17488199

[B36] Grogan B. F. Hsu J. R. Skeletal Trauma Research Consortium (2011). Volumetric muscle loss. J. Am. Acad. Orthop. Surg. 19 (Suppl. 1), S35–S37. 10.5435/00124635-201102001-00007 21304045

[B37] Haffner-Luntzer M. Hankenson K. D. Ignatius A. Pfeifer R. Khader B. A. Hildebrand F. (2019). Review of animal models of comorbidities in fracture-healing research. J. Orthop. Res. 37 (12), 2491–2498. 10.1002/jor.24454 31444806

[B38] Halloran B. P. Ferguson V. L. Simske S. J. Burghardt A. Venton L. L. Majumdar S. (2002). Changes in bone structure and mass with advancing age in the male C57BL/6J mouse. J. Bone Miner. Res. 17 (6), 1044–1050. 10.1359/jbmr.2002.17.6.1044 12054159

[B39] Handool K. O. Ibrahim S. M. Kaka U. Omar M. A. Abu J. Yusoff M. S. M. (2018). Optimization of a closed rat tibial fracture model. J. Exp. Orthop. 5 (1), 13 10.1186/s40634-018-0128-6 29721763 PMC5931953

[B40] Higginson B. K. (2009). Methods of running gait analysis. Curr. Sports Med. Rep. 8 (3), 136–141. 10.1249/JSR.0b013e3181a6187a 19436169

[B41] Hiltunen A. Vuorio E. Aro H. T. (1993). A standardized experimental fracture in the mouse tibia. J. Orthop. Res. 11 (2), 305–312. 10.1002/jor.1100110219 8483044

[B42] Hofman M. Kolejewska A. Greven J. Andruszkow H. Kobbe P. Tolba R. (2020). Gait analysis and muscle weight analysis after lower extremity fractures in a small animal model. Gait Posture 77, 207–213. 10.1016/j.gaitpost.2020.01.022 32058285

[B43] Holstein J. H. Matthys R. Histing T. Becker S. C. Fiedler M. Garcia P. (2009). Development of a stable closed femoral fracture model in mice. J. Surg. Res. 153 (1), 71–75. 10.1016/j.jss.2008.02.042 18656902

[B44] Holstein J. H. Menger M. D. Culemann U. Meier C. Pohlemann T. (2007). Development of a locking femur nail for mice. J. Biomech. 40 (1), 215–219. 10.1016/j.jbiomech.2005.10.034 16376352

[B45] Houdebine L. M. (2007). Transgenic animal models in biomedical research. Methods Mol. Biol. 360, 163–202. 10.1385/1-59745-165-7:163 17172731

[B46] Hu G. Zhang N. Li J. Wang J. Wu W. Li J. (2020). Tumor necrosis factor receptor associated factor 3 modulates cartilage degradation through suppression of interleukin 17 signaling. Am. J. Pathol. 190 (8), 1701–1712. 10.1016/j.ajpath.2020.04.016 32416098

[B47] Hurtgen B. J. Ward C. L. Garg K. Pollot B. E. Goldman S. M. McKinley T. O. (2016). Severe muscle trauma triggers heightened and prolonged local musculoskeletal inflammation and impairs adjacent tibia fracture healing. J. Musculoskelet. Neuronal Interact. 16 (2), 122–134. 27282456 PMC5114355

[B48] Jacenko O. Olsen B. R. (1995). Transgenic mouse models in studies of skeletal disorders. J. Rheumatol. Suppl. 43, 39–41. 7752133

[B49] Jain P. Chaney A. M. Carlson M. L. Jackson I. M. Rao A. James M. L. (2020). Neuroinflammation PET imaging: current opinion and future directions. J. Nucl. Med. 61 (8), 1107–1112. 10.2967/jnumed.119.229443 32620705 PMC7413228

[B50] Jepsen K. J. Silva M. J. Vashishth D. Guo X. E. van der Meulen M. C. (2015). Establishing biomechanical mechanisms in mouse models: practical guidelines for systematically evaluating phenotypic changes in the diaphyses of long bones. J. Bone Miner. Res. 30 (6), 951–966. 10.1002/jbmr.2539 25917136 PMC4794979

[B51] Jiang Y. Zhao J. White D. L. Genant H. K. (2000). Micro CT and Micro MR imaging of 3D architecture of animal skeleton. J. Musculoskelet. Neuronal Interact. 1 (1), 45–51. 15758525

[B52] Jimenez-Andrade J. M. Martin C. D. Koewler N. J. Freeman K. T. Sullivan L. J. Halvorson K. G. (2007). Nerve growth factor sequestering therapy attenuates non-malignant skeletal pain following fracture. Pain 133 (1-3), 183–196. 10.1016/j.pain.2007.06.016 17693023

[B53] Kalogeris T. Baines C. P. Krenz M. Korthuis R. J. (2012). Cell biology of ischemia/reperfusion injury. Int. Rev. Cell. Mol. Biol. 298, 229–317. 10.1016/B978-0-12-394309-5.00006-7 22878108 PMC3904795

[B54] Kappos E. A. Sieber P. K. Engels P. E. Mariolo A. V. D'Arpa S. Schaefer D. J. (2017). Validity and reliability of the CatWalk system as a static and dynamic gait analysis tool for the assessment of functional nerve recovery in small animal models. Brain Behav. 7 (7), e00723 10.1002/brb3.723 28729931 PMC5516599

[B55] Kaspar K. Schell H. Toben D. Matziolis G. Bail H. J. (2007). An easily reproducible and biomechanically standardized model to investigate bone healing in rats, using external fixation. Biomed. Tech. 52 (6), 383–390. 10.1515/BMT.2007.063 18047403

[B56] Kayal R. A. Alblowi J. McKenzie E. Krothapalli N. Silkman L. Gerstenfeld L. (2009). Diabetes causes the accelerated loss of cartilage during fracture repair which is reversed by insulin treatment. Bone 44 (2), 357–363. 10.1016/j.bone.2008.10.042 19010456 PMC2700945

[B57] Kelley N. Jeltema D. Duan Y. He Y. (2019). The NLRP3 inflammasome: an overview of mechanisms of activation and regulation. Int. J. Mol. Sci. 20 (13), 3328 10.3390/ijms20133328 PMC6651423 31284572

[B58] Koewler N. J. Freeman K. T. Buus R. J. Herrera M. B. Jimenez-Andrade J. M. Ghilardi J. R. (2007). Effects of a monoclonal antibody raised against nerve growth factor on skeletal pain and bone healing after fracture of the C57BL/6J mouse femur. J. Bone Miner. Res. 22 (11), 1732–1742. 10.1359/jbmr.070711 17638576

[B59] Krebs E. E. Carey T. S. Weinberger M. (2007). Accuracy of the pain numeric rating scale as a screening test in primary care. J. Gen. Intern. Med. 22 (10), 1453–1458. 10.1007/s11606-007-0321-2 17668269 PMC2305860

[B60] Kuhn B. N. Kalivas P. W. Bobadilla A. C. (2019). Understanding addiction using animal models. Front. Behav. Neurosci. 13, 262 10.3389/fnbeh.2019.00262 31849622 PMC6895146

[B61] Li Z. Helms J. A. (2021). Drill hole models to investigate bone repair. Methods Mol. Biol. 2221, 193–204. 10.1007/978-1-0716-0989-7_12 32979205

[B62] Li Z. Meyers C. A. Chang L. Lee S. Li Z. Tomlinson R. (2019). Fracture repair requires TrkA signaling by skeletal sensory nerves. J. Clin. Invest. 129 (12), 5137–5150. 10.1172/JCI128428 31638597 PMC6877307

[B63] Lichtor J. L. Zacny J. Korttila K. Apfelbaum J. L. Lane B. S. Rupani G. (1991). Alcohol after midazolam sedation: does it really matter? Anesth. Analg. 72 (5), 661–666. 10.1213/00000539-199105000-00016 2018224

[B64] Lin T. Kohno Y. Huang J. F. Romero-Lopez M. Maruyama M. Ueno M. (2019). Preconditioned or IL4-secreting mesenchymal stem cells enhanced osteogenesis at different stages. Tissue Eng. Part A. 25 (15-16), 1096–1103. 10.1089/ten.TEA.2018.0292 30652628 PMC6686696

[B65] Lin T. Kohno Y. Huang J. F. Romero-Lopez M. Pajarinen J. Maruyama M. (2018). NFκB sensing IL-4 secreting mesenchymal stem cells mitigate the proinflammatory response of macrophages exposed to polyethylene wear particles. J. Biomed. Mater. Res. 106 (10), 2744–2752. 10.1002/jbm.a.36504 PMC6207939 30084534

[B66] Lin T. Pajarinen J. Nabeshima A. Lu L. Nathan K. Yao Z. (2017). Establishment of NF-κB sensing and interleukin-4 secreting mesenchymal stromal cells as an "on-demand" drug delivery system to modulate inflammation. Cytotherapy 19 (9), 1025–1034. 10.1016/j.jcyt.2017.06.008 28739167 PMC5563472

[B67] Liodaki E. Wendlandt R. Waizner K. Schopp B. E. Mailänder P. Stang F. (2017). A biomechanical analysis of plate fixation using unicortical and bicortical screws in transverse metacarpal fracture models subjected to 4-point bending and dynamical bending test. Medicine (Baltimore). 96 (27), e6926 10.1097/MD.0000000000006926 28682860 PMC5502133

[B68] Loi F. Córdova L. A. Pajarinen J. Lin T. H. Yao Z. Goodman S. B. (2016). Inflammation, fracture and bone repair. Bone 86, 119–130. 10.1016/j.bone.2016.02.020 26946132 PMC4833637

[B69] Lu C. Miclau T. Hu D. Marcucio R. S. (2007). Ischemia leads to delayed union during fracture healing: a mouse model. J. Orthop. Res. 25 (1), 51–61. 10.1002/jor.20264 17019699 PMC2848995

[B70] Luna I. E. Kehlet H. Wede H. R. Hoevsgaard S. J. Aasvang E. K. (2019). Objectively measured early physical activity after total hip or knee arthroplasty. J. Clin. Monit. Comput. 33 (3), 509–522. 10.1007/s10877-018-0185-5 30039461

[B71] Lynch J. A. Silva M. J. (2008). *In vivo* static creep loading of the rat forelimb reduces ulnar structural properties at time-zero and induces damage-dependent woven bone formation. Bone 42 (5), 942–949. 10.1016/j.bone.2008.01.004 18295561 PMC2441934

[B72] Mackey I. G. Dixon E. A. Johnson K. Kong J. T. (2017). Dynamic quantitative sensory testing to characterize central pain processing. J. Vis. Exp. 120, 54452 10.3791/54452 PMC5407598 28287532

[B73] Macri F. Marques L. F. Backer R. C. Santos M. J. Belangero W. D. (2012). Validation of a standardised gait score to predict the healing of tibial fractures. J. Bone Joint Surg. Br. 94 (4), 544–548. 10.1302/0301-620X.94B4.27927 22434473

[B74] Manigrasso M. B. O'Connor J. P. (2010). Accelerated fracture healing in mice lacking the 5-lipoxygenase gene. Acta Orthop. 81 (6), 748–755. 10.3109/17453674.2010.533931 21067431 PMC3216088

[B75] Manigrasso M. B. O'Connor J. P. (2004). Characterization of a closed femur fracture model in mice. J. Orthop. Trauma. 18 (10), 687–695. 10.1097/00005131-200411000-00006 15507822

[B76] Manjavachi M. N. Motta E. M. Marotta D. M. Leite D. F. Calixto J. B. (2010). Mechanisms involved in IL-6-induced muscular mechanical hyperalgesia in mice. Pain 151 (2), 345–355. 10.1016/j.pain.2010.07.018 20709454

[B77] Mantyh P. W. Koltzenburg M. Mendell L. M. Tive L. Shelton D. L. (2011). Antagonism of nerve growth factor-TrkA signaling and the relief of pain. Anesthesiology 115 (1), 189–204. 10.1097/ALN.0b013e31821b1ac5 21602663 PMC3121917

[B78] Mantyh P. W. (2014). The neurobiology of skeletal pain. Eur. J. Neurosci. 39 (3), 508–519. 10.1111/ejn.12462 24494689 PMC4453827

[B79] Mark H. Bergholm J. Nilsson A. Rydevik B. Strömberg L. (2003). An external fixation method and device to study fracture healing in rats. Acta Orthop. Scand. 74 (4), 476–482. 10.1080/00016470310017820 14521302

[B80] Maruyama N. Shibata Y. Mochizuki A. Yamada A. Maki K. Inoue T. (2015). Bone micro-fragility caused by the mimetic aging processes in α-klotho deficient mice: *in situ* nanoindentation assessment of dilatational bands. Biomaterials 47, 62–71. 10.1016/j.biomaterials.2015.01.004 25682161

[B81] Masini B. D. Waterman S. M. Wenke J. C. Owens B. D. Hsu J. R. Ficke J. R. (2009). Resource utilization and disability outcome assessment of combat casualties from Operation Iraqi Freedom and Operation Enduring Freedom. J. Orthop. Trauma 23 (4), 261–266. 10.1097/BOT.0b013e31819dfa04 19318869

[B82] Merkle S. L. Sluka K. A. Frey-Law L. A. (2020). The interaction between pain and movement. J. Hand Ther. 33 (1), 60–66. 10.1016/j.jht.2018.05.001 30025839 PMC6335190

[B83] Meyer R. A. Jr. Desai B. R. Heiner D. E. Fiechtl J. Porter S. Meyer M. H. (2006). Young, adult, and old rats have similar changes in mRNA expression of many skeletal genes after fracture despite delayed healing with age. J. Orthop. Res. 24 (10), 1933–1944. 10.1002/jor.20124 16894589

[B84] Miclau K. R. Brazina S. A. Bahney C. S. Hankenson K. D. Hunt T. K. Marcucio R. S. (2017). Stimulating fracture healing in ischemic environments: does oxygen direct stem cell fate during fracture healing? Front. Cell Dev. Biol. 5, 45 10.3389/fcell.2017.00045 28523266 PMC5416746

[B85] Miller B. S. Bronk J. T. Nishiyama T. Yamagiwa H. Srivastava A. Bolander M. E. (2007). Pregnancy associated plasma protein-A is necessary for expeditious fracture healing in mice. J. Endocrinol. 192 (3), 505–513. 10.1677/JOE-06-0011 17332520

[B86] Mogil J. S. Pang D. S. J. Silva Dutra G. G. Chambers C. T. (2020). The development and use of facial grimace scales for pain measurement in animals. Neurosci. Biobehav. Rev. 116, 480–493. 10.1016/j.neubiorev.2020.07.013 32682741

[B87] Mumtaz H. Dallas M. Begonia M. Lara-Castillo N. Scott J. M. Johnson M. L. (2020). Age-related and sex-specific effects on architectural properties and biomechanical response of the C57BL/6N mouse femur, tibia and ulna. Bonekey Rep. 12, 100266 10.1016/j.bonr.2020.100266 PMC7215114 32420415

[B88] Muybridge E. (1979). Muybridge's Complete human and animal locomotion : all 781 plates from the 1887 Animal locomotion. New York, NY: Dover Publications.

[B89] Navratilova E. Porreca F. (2014). Reward and motivation in pain and pain relief. Nat. Neurosci. 17 (10), 1304–1312. 10.1038/nn.3811 25254980 PMC4301417

[B90] Neunaber C. Yesilkaya P. Pütz C. Krettek C. Hildebrand F. (2013). Differentiation of osteoprogenitor cells is affected by trauma-haemorrhage. Injury 44 (10), 1279–1284. 10.1016/j.injury.2013.05.011 23773407

[B91] Ngamkham S. Vincent C. Finnegan L. Holden J. E. Wang Z. J. Wilkie D. J. (2012). The McGill Pain Questionnaire as a multidimensional measure in people with cancer: an integrative review. Pain Manag. Nurs. 13 (1), 27–51. 10.1016/j.pmn.2010.12.003 22341138 PMC3285427

[B92] Osternig L. R. (1986). Isokinetic dynamometry: implications for muscle testing and rehabilitation. Exerc. Sport Sci. Rev. 14, 45–80. 3525192

[B93] Otto T. E. Patka P. Haarman H. J. (1995). Closed fracture healing: a rat model. Eur. Surg. Res. 27 (4), 277–284. 10.1159/000129410 7649215

[B94] Pathak S. Vachhani S. J. Jepsen K. J. Goldman H. M. Kalidindi S. R. (2012). Assessment of lamellar level properties in mouse bone utilizing a novel spherical nanoindentation data analysis method. J. Mech. Behav. Biomed. Mater. 13, 102–117. 10.1016/j.jmbbm.2012.03.018 22842281 PMC4786738

[B95] Pepe V. Oliviero S. Cristofolini L. Dall'Ara E. (2020). Regional nanoindentation properties in different locations on the mouse tibia from C57BL/6 and Balb/C female mice. Front. Bioeng. Biotechnol. 8, 478 10.3389/fbioe.2020.00478 32500069 PMC7243342

[B96] Percie du Sert N. Rice A. S. (2014). Improving the translation of analgesic drugs to the clinic: animal models of neuropathic pain. Br. J. Pharmacol. 171 (12), 2951–2963. 10.1111/bph.12645 24527763 PMC4055199

[B97] Poundarik A. A. Diab T. Sroga G. E. Ural A. Boskey A. L. Gundberg C. M. (2012). Dilatational band formation in bone. Proc. Natl. Acad. Sci. U.S.A. 109 (47), 19178–19183. 10.1073/pnas.1201513109 23129653 PMC3511118

[B98] Quarta M. Cromie Lear M. J. Blonigan J. Paine P. Chacon R. Rando T. A. (2018). Biomechanics show stem cell necessity for effective treatment of volumetric muscle loss using bioengineered constructs. NPJ Regen. Med. 3, 18 10.1038/s41536-018-0057-0 30323949 PMC6180087

[B99] Quarta M. Cromie M. Chacon R. Blonigan J. Garcia V. Akimenko I. (2017). Bioengineered constructs combined with exercise enhance stem cell-mediated treatment of volumetric muscle loss. Nat. Commun. 8, 15613 10.1038/ncomms15613 28631758 PMC5481841

[B100] Rosenbaum D. Macri F. Lupselo F. S. Preis O. C. (2014). Gait and function as tools for the assessment of fracture repair - the role of movement analysis for the assessment of fracture healing. Injury 45 (Suppl. 2), S39–S43. 10.1016/j.injury.2014.04.007 24857027

[B101] Sakai R. Miyasaka K. Minagawa E. Ohtsuka T. Harada A. Yoshikawa Y. (2008). A minute bone bending angle measurement method using echo-tracking for assessment of bone strength *in vivo* . IEEE Ultrason. Symp., 241–244. 10.1109/ULTSYM.2008.0059

[B102] Schäfers M. Sorkin L. S. Sommer C. (2003). Intramuscular injection of tumor necrosis factor-alpha induces muscle hyperalgesia in rats. Pain 104 (3), 579–588. 10.1016/s0304-3959(03)00115-5 12927630

[B103] Schindeler A. Morse A. Harry L. Godfrey C. Mikulec K. McDonald M. (2008). Models of tibial fracture healing in normal and Nf1-deficient mice. J. Orthop. Res. 26 (8), 1053–1060. 10.1002/jor.20628 18383150

[B104] Schmidhammer R. Zandieh S. Mittermayr R. Pelinka L. E. Leixnering M. Hopf R. (2006). Assessment of bone union/nonunion in an experimental model using microcomputed technology. J. Trauma 61 (1), 199–205. 10.1097/01.ta.0000195987.57939.7e 16832271

[B105] Schriefer J. L. Robling A. G. Warden S. J. Fournier A. J. Mason J. J. Turner C. H. (2005). A comparison of mechanical properties derived from multiple skeletal sites in mice. J. Biomech. 38 (3), 467–475. 10.1016/j.jbiomech.2004.04.020 15652544

[B106] Seebeck P. Thompson M. S. Parwani A. Taylor W. R. Schell H. Duda G. N. (2005). Gait evaluation: a tool to monitor bone healing? Clin. Biomech. 20 (9), 883–891. 10.1016/j.clinbiomech.2005.05.010 16009475

[B107] Shepherd A. J. Mickle A. D. Golden J. P. Mack M. R. Halabi C. M. de Kloet A. D. (2018). Macrophage angiotensin II type 2 receptor triggers neuropathic pain. Proc. Natl. Acad. Sci. U. S. A. 115 (34), E8057–E8066. 10.1073/pnas.1721815115 30082378 PMC6112686

[B108] Shepherd A. J. Mohapatra D. P. (2018). Pharmacological validation of voluntary gait and mechanical sensitivity assays associated with inflammatory and neuropathic pain in mice. Neuropharmacology 130, 18–29. 10.1016/j.neuropharm.2017.11.036 29191755 PMC5743638

[B109] Shi X. Guo T. Z. Li W. Sahbaie P. Rice K. C. Sulima A. (2018). Exercise reverses nociceptive sensitization, upregulated neuropeptide signaling, inflammatory changes, anxiety, and memory impairment in a mouse tibia fracture model. Anesthesiology 129 (3), 557–575. 10.1097/ALN.0000000000002332 29994924 PMC6092202

[B110] Sicari B. M. Rubin J. P. Dearth C. L. Wolf M. T. Ambrosio F. Boninger M. (2014). An acellular biologic scaffold promotes skeletal muscle formation in mice and humans with volumetric muscle loss. Sci. Transl. Med. 6 (234), 234ra58 10.1126/scitranslmed.3008085 PMC5942588 24786326

[B111] Solomon L. B. Stevenson A. W. Callary S. A. Sullivan T. R. Howie D. W. Chehade M. J. (2010). The accuracy and precision of radiostereometric analysis in monitoring tibial plateau fractures. Acta Orthop. 81 (4), 487–494. 10.3109/17453674.2010.487930 20465528 PMC2917573

[B112] Stojadinovic A. Auton A. Peoples G. E. McKnight G. M. Shields C. Croll S. M. (2006). Responding to challenges in modern combat casualty care: innovative use of advanced regional anesthesia. Pain Med. 7 (4), 330–338. 10.1111/j.1526-4637.2006.00171.x 16898944

[B113] Sturgeon J. A. Darnall B. D. Kao M. C. Mackey S. C. (2015a). Physical and psychological correlates of fatigue and physical function: a Collaborative Health Outcomes Information Registry (CHOIR) study. J. Pain 16(3), 291–298. 10.1016/j.jpain.2014.12.004 25536536 PMC4352393

[B114] Sturgeon J. A. Dixon E. A. Darnall B. D. Mackey S. C. (2015b). Contributions of physical function and satisfaction with social roles to emotional distress in chronic pain: a Collaborative Health Outcomes Information Registry (CHOIR) study. Pain. 156 (12), 2627–2633. 10.1097/j.pain.0000000000000313 26230739 PMC4936835

[B115] Tawfik V. L. Huck N. A. Baca Q. J. Ganio E. A. Haight E. S. Culos A. (2020a). Systematic immunophenotyping reveals sex-specific responses after painful injury in mice. Front. Immunol. 11, 1652 10.3389/fimmu.2020.01652 32849569 PMC7403191

[B116] Tawfik V. L. Quarta M. Paine P. Forman T. E. Pajarinen J. Takemura Y. (2020b). Angiotensin receptor blockade mimics the effect of exercise on recovery after orthopaedic trauma by decreasing pain and improving muscle regeneration. J. Physiol. 598 (2), 317–329. 10.1113/JP278991 31784993 PMC8830491

[B117] Vennin S. Desyatova A. Turner J. A. Watson P. A. Lappe J. M. Recker R. R. (2017). Intrinsic material property differences in bone tissue from patients suffering low-trauma osteoporotic fractures, compared to matched non-fracturing women. Bone. 97, 233–242. 10.1016/j.bone.2017.01.031 28132909 PMC5367951

[B118] Wang H. Spinner R. J. Sorenson E. J. Windebank A. J. (2008). Measurement of forelimb function by digital video motion analysis in rat nerve transection models. J. Peripher. Nerv. Syst. 13 (1), 92–102. 10.1111/j.1529-8027.2008.00162.x 18346235

[B119] Watanabe K. Hishiya A. (2005). Mouse models of senile osteoporosis. Mol. Aspects Med. 26 (3), 221–231. 10.1016/j.mam.2005.01.006 15811436

[B120] Williams J. M. Zurawski J. Mikecz K. Glant T. T. (1993). Functional assessment of joint use in experimental inflammatory murine arthritis. J. Orthop. Res. 11 (2), 172–180. 10.1002/jor.1100110204 8483030

[B121] Willinghamm M. D. Brodt M. D. Lee K. L. Stephens A. L. Ye J. Silva M. J. (2010). Age-related changes in bone structure and strength in female and male BALB/c mice. Calcif. Tissue Int. 86 (6), 470–483. 10.1007/s00223-010-9359-y 20405109 PMC2895262

[B122] Wong R. M. Y. Li T. K. Li J. Ho W. T. Chow S. K. Leung S. S. Y. (2020). A systematic review on current osteosynthesis-associated infection animal fracture models. J. Orthop. Translat. 23, 8–20. 10.1016/j.jot.2020.03.002 32440511 PMC7231979

[B123] Woolf C. J. (2010). Overcoming obstacles to developing new analgesics. Nat. Med. 16 (11), 1241–1247. 10.1038/nm.2230 20948534

[B124] Yousefzadeh N. Kashfi K. Jeddi S. Ghasemi A. (2020). Ovariectomized rat model of osteoporosis: a practical guide. EXCLI J. 19, 89–107. 10.17179/excli2019-1990 32038119 PMC7003643

[B125] Zhang N. Chim Y. N. Wang J. Wong R. M. Y. Chow S. K. H. Cheung W. H. (2020). Impaired fracture healing in sarco-osteoporotic mice can Be rescued by vibration treatment through myostatin suppression. J. Orthop. Res. 38 (2), 277–287. 10.1002/jor.24477 31535727

[B126] Zhang N. Chow S. K. H. Leung K. S. Lee H. H. Cheung W. H. (2017). An animal model of co-existing sarcopenia and osteoporotic fracture in senescence accelerated mouse prone 8 (SAMP8). Exp. Gerontol. 97, 1–8. 10.1016/j.exger.2017.07.008 28711604

[B127] Zwingenberger S. Niederlohmann E. Vater C. Rammelt S. Matthys R. Bernhardt R. (2013). Establishment of a femoral critical-size bone defect model in immunodeficient mice. J. Surg. Res. 181 (1), e7–e14. 10.1016/j.jss.2012.06.039 22765996 PMC4045639

